# Socialisation and its effect on play behaviour and aggression in the domestic pig (*Sus scrofa*)

**DOI:** 10.1038/s41598-019-40980-1

**Published:** 2019-03-12

**Authors:** Jennifer E. Weller, Irene Camerlink, Simon P. Turner, Marianne Farish, Gareth Arnott

**Affiliations:** 1 Institute for Global Food Security, School of Biological Sciences, Queens University Belfast, Belfast, UK; 2 Institute of Animal Husbandry and Animal Welfare, University of Veterinary Medicine Vienna (Vetmeduni), Vienna, Austria; 3 Animal Behaviour & Welfare, Scotland’s Rural College (SRUC), Edinburgh, UK

## Abstract

There is considerable interest in how early life experiences shape behavioural development. For example, the socialisation of unfamiliar pigs pre-weaning has been suggested to decrease aggression during later life. However, the behavioural mechanisms behind this socialisation effect remain unexplored. We allowed 12 litters of domestic pigs (*Sus scrofa*) to move freely between their home pen and a neighbouring pen (socialisation) during the lactation period, while keeping 12 litters isolated in their home pen (control). Contrary to predictions, socialisation did not result in higher levels of social play. However, control individuals engaged in more sow directed play than those that underwent socialisation. Consistent with predictions, males performed more piglet directed play than females. Social play behaviour pre-weaning was found to be highly concordant within individuals from both treatments. Post-weaning, 148 pigs were selected to perform two resident-intruder tests to assay aggressiveness. As predicted, socialised individuals were quicker to attack than controls, although females were more aggressive than males. Additionally, play fighting experience was found to negatively correlate with attack latency in females, supporting the hypothesis that early-life play experience is likely to be sexually dimorphic when males and females show pronounced differences in their later-life social behaviour.

## Introduction

Under free-range conditions the weaning of domestic pigs (*Sus scrofa*) is a gradual process that can occur anywhere between 8–15 weeks after farrowing^1^. However, on a commercial farm profitability is maximized when weaning is artificially induced at around 4 weeks of age^2,3^. Early weaning results in a number of stressors for piglets, including sudden separation from the sow, relocation to a new environment, abrupt dietary changes, and the introduction of unfamiliar individuals^4^. As such, early weaning has been reported to result in increased vocalisations^5,6^, decreased play behaviour^7^, elevated plasma cortisol concentrations^8^, decreased average daily weight gain^9^, and increased aggression^10^.

Often first occurring immediately after weaning, the mixing of unfamiliar individuals is a common practice on most commercial pig farms^11–13^ and has repeatedly been shown to cause a peak in aggressive behaviour for up to 48 hours while a new social hierarchy is established^14,15^. This can have long-term negative effects on livestock productivity and welfare^16^ due to decreases in food intake and weight gain, the potential for injury, and in extreme cases, increased mortality^11,12^. A variety of aggression-reducing methods have been investigated^17^, including the use of odour masking agents^18,19^, the provision of escape hides^20^, and the mixing of pigs in a novel, dark pen containing ample straw^14^. However, none of these methods has proven completely successful^12,17^.

Previously, studies considering the effect of pre-weaning environment on post-mixing aggression have found that individuals from an enriched environment appear to form dominance relationships more easily than those reared in a barren environment^21,22^. In order to isolate out the aspect of environmental enrichment causing this effect, multiple studies have explored the influence of the early-life social environment on aggressiveness by allowing piglets to interact freely with non-littermates pre-weaning^23,24^. Socialised individuals were quicker than controls to perform aggressive behaviour when they were later regrouped after weaning but showed less aggression on the second day post-regrouping, suggesting that stable dominance relationships were established more rapidly^24^. Subsequently investigations have also reported a positive effect of socialisation on agonistic behaviour, with individuals seeming to engage in fewer, shorter and less intensive fighting bouts than those of control individuals^25–27^.

One possible explanation for this is that early-life socialisation closely resembles the natural social environment of both free-ranging domestic pigs and wild boars^24^. Typically, under natural conditions, piglets are introduced to unfamiliar conspecifics at 1–2 weeks of age, when sows abandon their isolated farrowing nests and re-join the social group^28,29^. Sows will often follow each other throughout the day and sleep together at night, providing numerous opportunities for non-littermate piglets of the same age to interact^16^. Furthermore, free-range piglets have shown a strong age-dependant motivation to interact socially around this time^16^. During this period, the frequency of play behaviour is also at its highest^30^, suggesting a potential link between socialisation and play behaviour.

Many researchers have suggested that play is a good indicator of an animal’s welfare^31,32^ (but see^33^) as it is only performed when all other primary needs have been satisfied^30,34^. As such, play behaviour is often most common in young animals under the care of adult conspecifics^30^. Many benefits of early-life play experience have been proposed, including training for unexpected situations both cognitively and physically (training for the unexpected hypothesis^35^), the facilitation of skeletal muscle fibre differentiation (motor training hypothesis^36^), the building of social bonds between individuals (social cohesion hypothesis^37,38^), and the development of social skills required for later-life social interactions^34,39^. Such skills may include the recognition and assessment of other individuals, as well as the motor skills required to engage in cooperative or competitive behaviours^40^. Additionally, play fighting may also provide individuals with a reference regarding their own fighting ability, in the same way that individuals are suggested to infer information about their own fighting ability from the outcomes of previous aggressive conflicts^41^. Play may therefore serve as the linking factor between the early-life socialisation of pigs and the subsequent decreases in post-weaning aggressive behaviour that have been reported. However, this remains to be explored. Here we therefore investigated the effect of early-life socialisation on pre-weaning social play experience using a variety of measures and examine the association between engagement in play and later aggressive behaviour.

Three key hypotheses were explored regarding the role of the early-life social environment on pre-weaning play behaviour and subsequent post-weaning aggression: 1) that males would engage in more social play than females, 2) that individuals undergoing socialisation with non-littermates pre-weaning would experience more social play than their control counterparts, and 3) that the greater social play experience of socialised individuals pre-weaning would result in a shorter latency to attack a subordinate individual during RI testing. It was hypothesised that socialised piglets would engage in more instances of social play (particularly play fighting), than their non-socialised controls, while males would engage in more play fights than females, given the observed sexual dimorphism in the post-weaning social behaviour of wild boar^42^.

Previously, it has been suggested that if social play experience aids in future social interactions, sex differences in play behaviour should be observed when adult males and females differ in their later-life social environment^39,42–45^ (but see^46^). For example, in many species intrasexual selection has resulted in males performing more intense aggressive behaviour than females^47^. As such, sex differences in juvenile play are expected to resemble variations in male and female reproductive strategies^48^. Reinhardt *et al*.^49^ found that pre-pubertal male bovine calves (*Bos indicus*) performed more play mounting and pushing than females, while both sexes preferred to direct play behaviours towards male individuals. Sex differences in play behaviour have also been observed in a range of mammals, including wild^48^ and domestic lambs^50^ (*Ovis candien*, *Ovis aries*), Siberia ibex^51^ (*Capra ibex sybirica*), Steller sea lions^52^ (*Eumetopias jubatus*), domestic dogs^53^ (*Canis lupus familiaris*), and horses^45,54^ (*Equus caballus*). In all of these species, adult males typically engage in more severe aggression than females and were found to perform or initiate greater amounts of juvenile social play. However, studies looking at sexual dimorphism in domestic pig play behaviour have been inconclusive^13,30,40,55,56^, despite the high risk of physical injury associated with male-male fights during the breeding season. Differences in reported results are likely to be due to variation in the ethograms used to define play, the age of the pigs at testing, and the number of pigs studied. Here we aim to specifically identify the extent to which males and females differ in their pre-weaning experience of play fighting, other forms of piglet-directed play, and sow-directed play.

Additionally, Resident-Intruder Tests (hereafter RI tests) were carried out post-weaning at 7 weeks of age using both socialised and control individuals in order to investigate the effects of both early-life play experience and social environment on post-weaning aggression^57,58^. The latency of a superior individual (i.e. the resident) to attack an obviously subordinate conspecific (i.e. the intruder) is considered to be a quantifiable and repeatable measure of the attackers aggressiveness^59^, and was therefore measured once per day on two consecutive days. It was predicted that increased play experience would result in a decreased attack latency due to an improved recognition of an inferior intruder. Therefore, if early-life social environment influences social play experience, socialised individuals were expected to demonstrate a shorter attack latency than controls.

## Methods

### Ethical Note

This study was carried out in accordance with the recommended European Guidelines for the accommodation and care of animals, the UK Government DEFRA animal welfare codes, and the ASAB/ABS guidelines. All procedures were approved by the UK Government Home Office (Project licence PPL60/4330) under the Animals Scientific Procedures Act 1986 and SRUC’s Animal Ethics Committee (no. ED RP 04–2014), and were performed in constant collaboration with SRUC’s named veterinary surgeon. No detrimental health effects of socialisation were observed over the course of this experiment. RI tests were ended immediately after the onset of an attack in order to prevent injury.

### Animals and Housing

**Figure 1:**
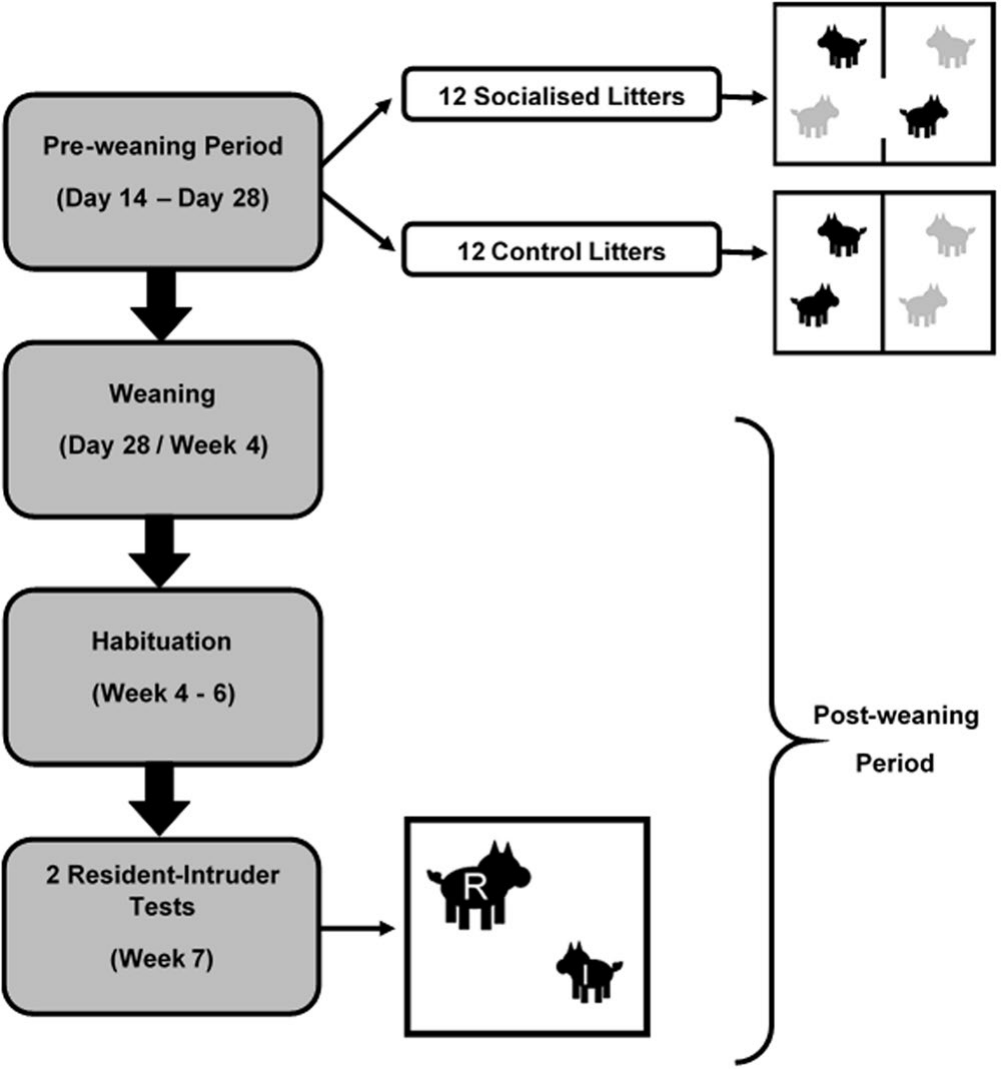
Experimental design. 12 litters were socialised by removing part of the pen divider. This allowed them access to a neighbouring pen and subsequently similarly aged non-littermates. 12 litters were kept isolated within their home pen as is standard procedure on most domestic pig farms. After weaning at day 28 piglets were habituated to human contact and to test conditions. At week 7, selected individuals (residents) were introduced to 2 novel, inferior piglets (intruders) over the course of 2 Resident-Intruder tests performed over consecutive days in order to record attack latency.

Data for this study were collected at SRUC Easter Howgate pig unit (Roslin, Scotland) between January and October 2016. Piglets of 24 Large White x Landrace sows inseminated with American Hampshire semen were studied over two batches. Pre-weaning play behaviour was collected for a total of 263 individuals (138 males, 125 females) from 24 litters. For each batch, 6 of the 12 litters were selected to undergo the socialisation treatment, while the remaining 6 litters served as a control (Experimental design; Fig. 1). This resulted in play experience data being available for a total of 135 socialised piglets (69 males, 66 females) and 128 control piglets (68 males, 60 females). Five days before parturition sows were allocated to separate farrowing pens (1.50 m × 2.50 m) containing a conventional farrowing crate (0.55 m × 2.25 m). Piglets had access to an additional 0.65 m × 1.50 m heated creep located directly in front of the sow’s crate. Heated creeps were warmed to a constant 35 °C using an underfloor system.

### Socialisation

Socialisation was performed by removing the solid partition separating two neighbouring farrowing pens and replacing it with a new barrier containing a ~35 × 74 cm opening positioned between the middle and the rear of the sow. This allowed piglets to access both pens but still prevented neighbouring sows from being able to see or interact with one another. Barriers were switched on day 14 postpartum and remained *in situ* until weaning at day 28. The remaining control litters were kept isolated within their home pen as is standard practice on most commercial pig farms. Although the socialisation procedure resulted in an increase in total available space, piglet density remained consistent between the two treatments.

### Collection of Play Data

In order to calculate social play experience, all socialised and control pens were filmed using Geovision surveillance hardware linked to GV-1480 playback software between 10:00 AM and 16:00 PM on a range of days across the pre-weaning period (i.e. days 14, 16, 19, 21, 24, and 26 after birth). Footage taken during the first 15 minutes of every hour was played back using EZViewLog500 and all instances of social play were coded using a clearly defined ethogram (see Supplementary Table S1). The I.D of the initiator individual (the piglet that began the interaction), the I.D of the target individual (the piglet that was subjected to the interaction), and, if appropriate, the target individual’s response (accept or reject play fighting invite) were recorded. From this, 3 frequency measures of social play experience were calculated for each piglet on a daily basis; play fighting experience (sum of all successful play fighting invitations performed and all play fighting invitations accepted), additional piglet directed play (total number of nudges, chases, and mounts performed), and sow directed play (all attempts to climb upon or make naso-naso contact with the sow). All observations were made by one observer.

### Weaning and Habituation

Piglets were weaned on day 28 by removing the sow from the farrowing crate as is standard practice at Easter Howgate pig unit. All pigs were weighed and tagged. Socialised litters were separated from non-littermates and a total of 92 socialised (52 males, 40 female) and 60 control (34 males, 26 females) individuals were selected based on body weight to perform RI tests as resident individuals. The remaining individuals were used as intruders.

All pigs were moved to another building where they were placed in 1.90 m × 5.80 m pens containing a 4 space feeder, a drinker and straw bedding throughout. Pens also contained a division gate (located half way down the pen) which could be shut to create a home pen arena (1.90 m × 2.90 m) during resident-intruder testing. Pigs were habituated to their new environment and to human interactions before gradually being habituated to spending short amounts of time (up 3 minutes) in the test arena. Group size was gradually reduced until pigs were comfortable spending a short time alone in the arena.

### Resident-Intruder Tests

RI tests were conducted at 7 weeks of age as a repeatable means of quantifying aggressiveness^59,60^. Once the resident was isolated within the familiar home pen arena, an unfamiliar intruder piglet of ~75% (mean ± SE = 76.89 ± 0.64%) of the resident’s body weight was introduced, creating an asymmetrical scenario in which the resident was likely to attack the inferior intruder. Once contact had been made, the latency of the resident to attack the intruder, i.e. the time until the resident bit the intruder, was recorded. Tests were concluded when either pig was observed attacking the other, 5 minutes after first contact with no subsequent physical aggression, if one of the individuals was mounted a total of 5 times, or if either the resident or intruder showed fear behaviour (escape attempt or repeated vocalizations). If no aggression was observed within 5 minutes, or if the test was ended due to mounting/escape attempts, residents were given the maximum attack latency score of 300 seconds. In a minority of tests (16%) the intruder was the first to attack. In such cases, the resident was given the maximum attack latency score, unless it retaliated immediately (n = 15/48), in which case attack latency was considered to be the difference between the time of first contact and retaliation. Residents were tested twice over two consecutive days using two different intruders, resulting in data being collected for a total of 304 RI tests. Once a test was ended, intruders were returned to their home pen and the dividing gate was reopened, allowing the resident to re-join its littermates.

### Statistical Analysis

One control litter was excluded from the analysis due to technical difficulties with video footage. Additionally, one female from the control treatment was humanely euthanized before weaning due to health concerns unrelated to the trial and was subsequently removed from the analysis. This reduced the available data on the play experience of control individuals to 119 pigs (64 males, 55 females) and 56 pigs (32 males, 24 females) for attack latency. No socialised pigs were excluded. All data analysis was performed in the statistical package R version 3.4.0 (The R Foundation for Statistical Computing). Data are presented as means ± standard error.

Analysis of the response variables play fighting, additional piglet directed play, and sow directed play was performed using separate general linear mixed effect models (GLME’s) containing treatment (socialised versus control), sex, and day of recording (14, 16, 19, 21, 23, 26) as fixed effects. Interaction effects were also included. Batch, litter ID, and piglet ID were further included in the model as nested random factors to account for the non-independence of piglets across days, as well as litter effects^56^. The residuals of all models were checked for normality and count data were square-root transformed when appropriate. Models initially included all relevant explanatory factors and interactions. These were then removed using a top-down approach in order to increase the fit of the model to the data, as assessed through Akaike’s information criterion (AIC) values. Models were fitted using maximum likelihood (ML) and the best model was examined by means of a Wald’s test using restricted maximum likelihood (REML). Multiple comparisons of means using Tukey contrasts was employed post-hoc to further explore variation in play behaviour between days of recording. Kendall’s Coefficient of Concordance was used to examine the consistency of individual social play behaviours across the observation period, both in general and within each treatment^61^. Piglet preference for littermate or non-littermate play partners was examined using individuals from the socialised treatment. Partner choice for play fighting and additional piglet directed play was explored using GLME’s containing partner relationship (littermate versus non-littermate), sex, and day of observation as fixed effects, and batch, litter ID, and piglet ID as nested random factors.

The influence of socialisation on piglet attack latency was examined using another GLME containing treatment, sex, day of RI testing, and total play fighting observed as fixed effects and batch, litter, and piglet ID as nested random effects. Model residuals were found to be normal and therefore attack latency was not transformed. Explanatory variables and interaction effects were again sequentially removed in order to maximise the model’s fit to the data.

## Results

### Play Experience

**Figure 2:**
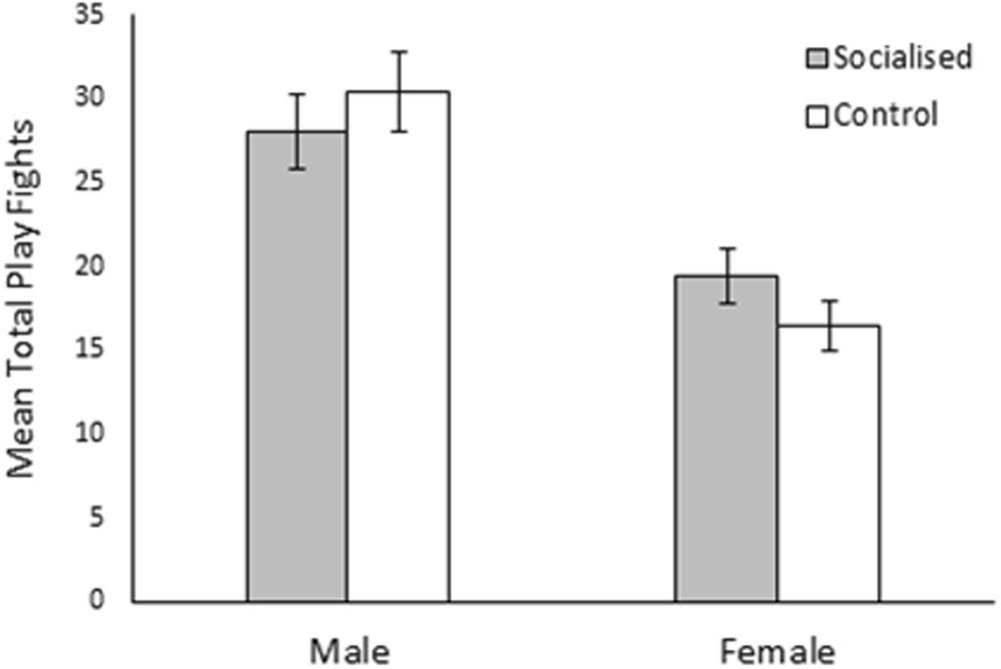
Effect of socialisation on the total number of play fights that male and female pigs engaged in over the pre-weaning observation period. Error bars represent the standard error of the mean.

As predicted, males engaged in more play fighting bouts than females over the pre-weaning observation period (males: 29.22 ± 1.61, females: 18.11 ± 1.14; χ^2^_1_ = 31.41, *p* < 0.001; Fig. 2). However, no significant effect of treatment was observed (socialised: 23.87 ± 1.43, control: 23.99 ± 1.58; χ^2^_1_ = 0.002, *p* = 0.961). Day was found to affect the level of play fighting observed (χ^2^_1_ = 14.70, *p* = 0.012) although only day 19 and 24 significantly differed from each other (*z* = −3.47, *p* = 0.007), with more play fighting occurring on day 19 (4.34 ± 0.02) than day 24 (3.62 ± 0.02). No significant interaction effects were observed (*p* > 0.05). Furthermore, play fighting behaviour was found to be highly concordant over the observation period (W_253_ = 0.44, *p* < 0.001). There was little difference in the level of concordance observed in the socialisation (W_134_ = 0.42, *p* < 0.001) and control (W_118_ = 0.45, *p* < 0.001) treatments.

**Figure 3:**
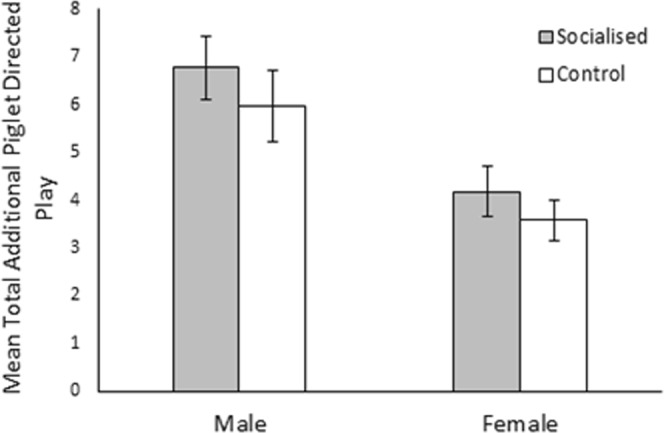
Effect of socialisation on the total amount of additional piglet directed play bouts that male and female pigs performed during the pre-weaning observation period. Error bars represent the standard error of the mean.

**Figure 4:**
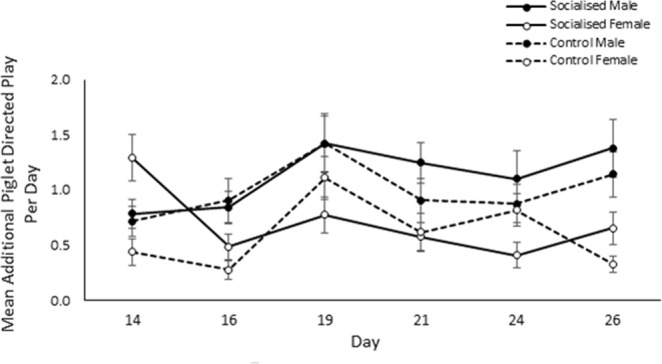
Additional piglet directed play according to observation day and treatment. Error bars represent the standard error of the mean.

Males engaged in significantly more additional piglet directed play than females (males: 6.38 ± 0.51, females: 3.91 ± 0.35; χ^2^_1_ = 19.14, *p* < 0.001; Fig. 3). Furthermore, interaction effects revealed that additional piglet directed play significantly differed between days (χ^2^_5_ = 28.74, *p* < 0.001) with the extent varying between treatments (χ^2^_5_ = 18.26, *p* = 0.003) and sex (χ^2^_5_ = 12.20, *p* = 0.032). However, no clear pattern could be identified (see Fig. 4). Once again, additional piglet directed play as observed across the pre-weaning period was found to be highly concordant (W_253_ = 0.33, *p* < 0.001), regardless of treatment (socialised: W_134_ = 0.29 *p* < 0.001, control: W_118_ = 0.39, *p* < 0.001).

**Figure 5:**
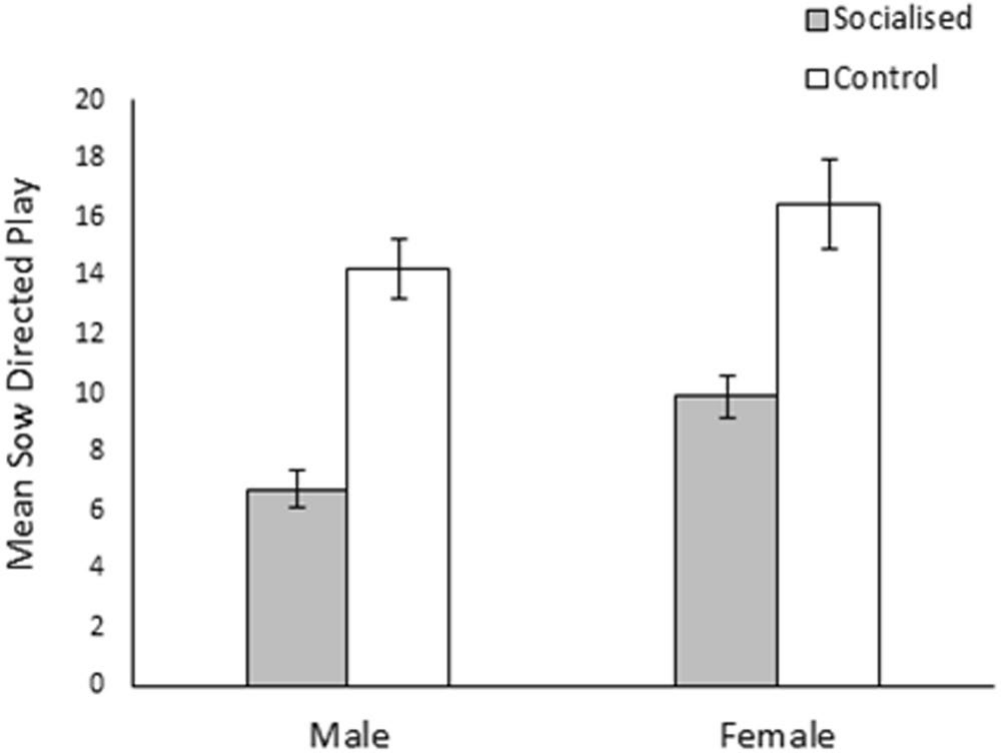
Effect of socialisation on the total amount of sow directed play that male and female pigs performed during the pre-weaning observation period. Error bars represent the standard error of the mean.

**Figure 6:**
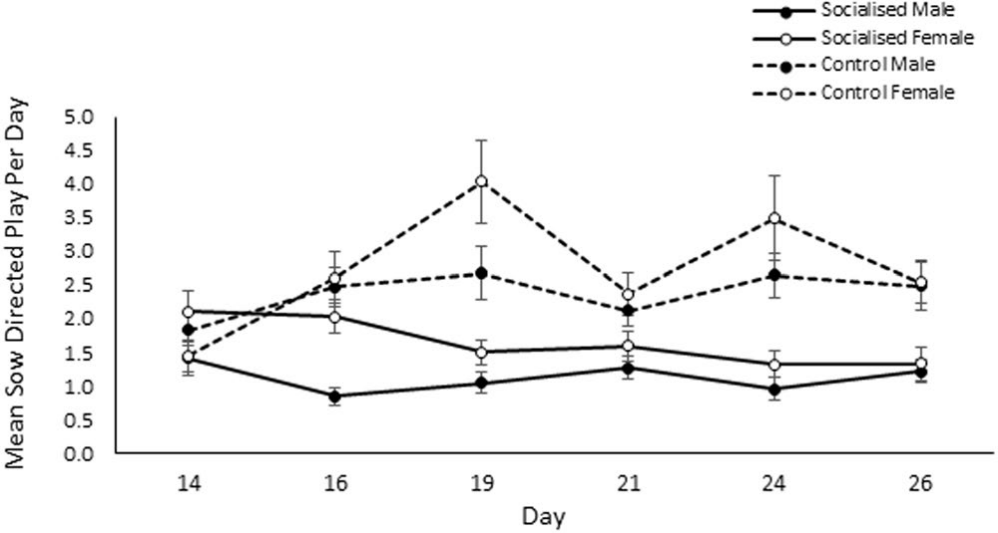
Sow directed play according to observation day and treatment. Error bars represent the standard error of the mean.

Females on the other hand engaged in more sow directed play than males (males: 10.33 ± 0.68, females: 12.88 ± 0.85; χ^2^_1_ = 4.75, *p* = 0.029; Fig. 5) while control pigs performed more sow directed play than those that had been socialised (socialised: 8.27 ± 0.50, control: 15.27 ± 0.90; χ^2^_1_ = 13.04, *p* < 0.001). An interaction effect of day and treatment was also observed (χ^2^_5_ = 31.52, *p* < 0.001; see Fig. 6) although post hoc testing revealed that only day 14 (1.70 ± 0.13) significantly differed from day 19 (2.22 ± 0.19; *z* = 5.12, *p* < 0.001) and day 24 (2.02 ± 0.18; *z* = 3.95, *p* = 0.001). Sow directed play behaviour was again found to be highly concordant across the duration of the observation period (W_253_ = 0.36, *p* < 0.001.) with neither treatment strongly differing in concordance (socialised: W_134_ = 0.33, *p* < 0.001, control: W_118_ = 0.32, *p* < 0.001).

**Figure 7:**
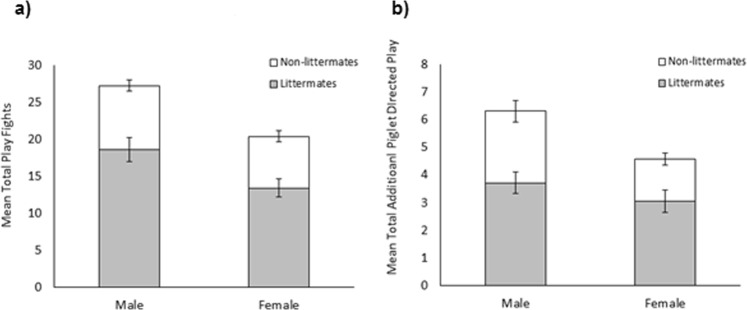
Partner preference of socialised individuals for (**a**) play fighting and (**b**) piglet directed play. Grey areas represent the average total play performed with littermates. White areas represent the average total play performed with non-littermates. The combined height of the bars shows the average total play performed by either sex. Error bars represent standard errors of the mean.

**Figure 8:**
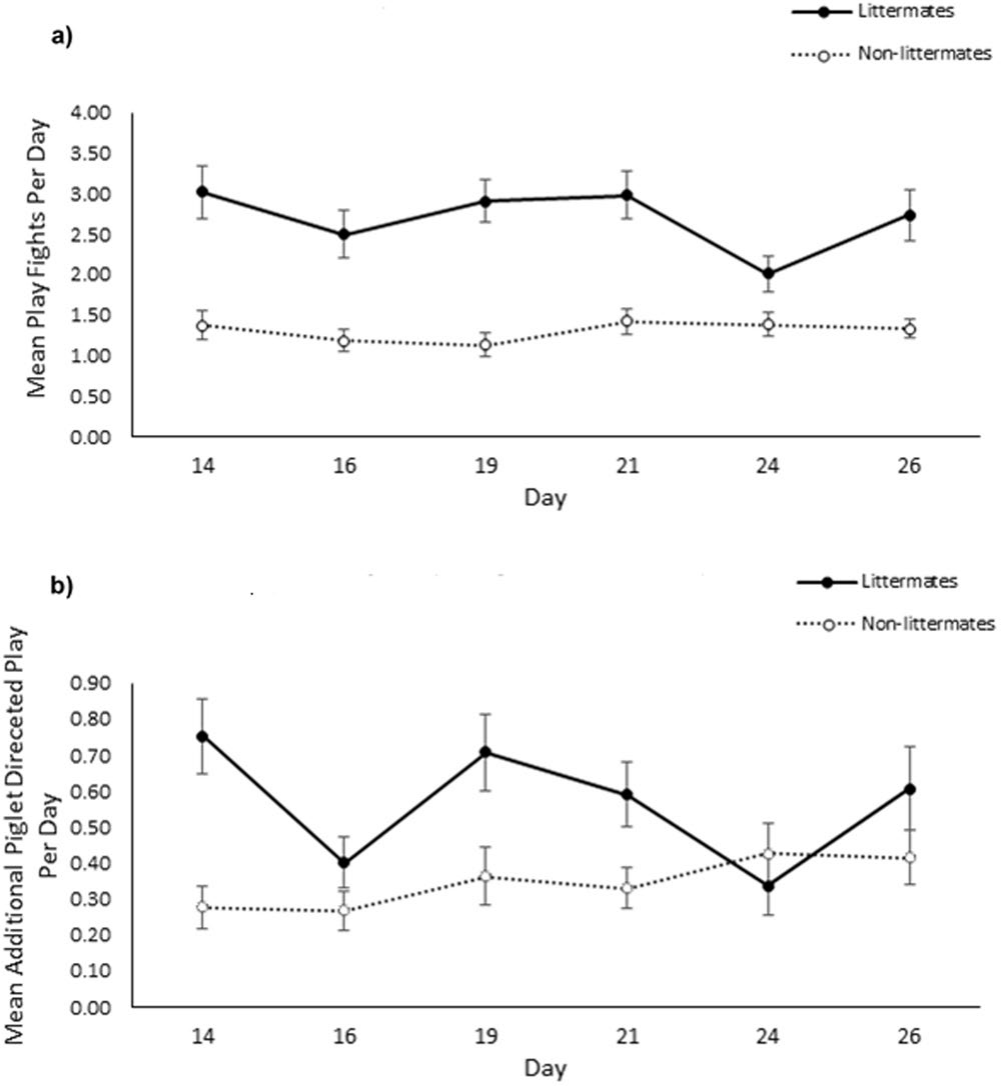
Partner preference of socialised individuals for (**a**) play fighting and (**b**) piglet directed play across the pre-weaning observation period. Error bars represent standard errors of the mean.

Socialised individuals showed a significant preference for littermate partners when engaging in both play fighting (χ^2^_1_ = 124.36, *p* < 0.001) and additional piglet directed play (χ^2^_1_ = 24.46, *p* < 0.001), performing approximately a third of these interactions with non-littermates (play fighting: 33.4 ± 1.6%, additional piglet directed play: 31.6 ± 2.4%; Fig. 7). Furthermore, as expected from previous results, the play fighting experience (χ^2^_1_ = 5.84, *p* = 0.016) and the amount of additional piglet directed play performed (χ^2^_1_ = 5.59, *p* = 0.018) by socialised individuals were significantly influenced by sex. In addition, a two-way interaction effect of sex and day was found to influence the additional piglet directed play of socialised individuals (χ^2^_5_ = 15.85, *p* = 0.007), although no clear pattern could be observed (Fig. 8*)*. Day of observation did not significantly influence either measure of social play behaviour (play fighting: χ^2^_5_ = 10.36, *p* = 0.066, additional piglet directed play: χ^2^_5_ = 10.40, *p* = 0.065, Fig. 8), however the amount of additional piglet directed play socialised individuals directed towards non-litters mates tended to increase over the observation period. A significant interaction effect of play partner relationship and day of observation on play fighting experience was observed (χ^2^_1_ = 11.91, *p* = 0.04), suggesting that the extent to which individuals preferred play fighting with littermates varied between days. However, no clear pattern could be identified.

### Attack Latency

**Figure 9:**
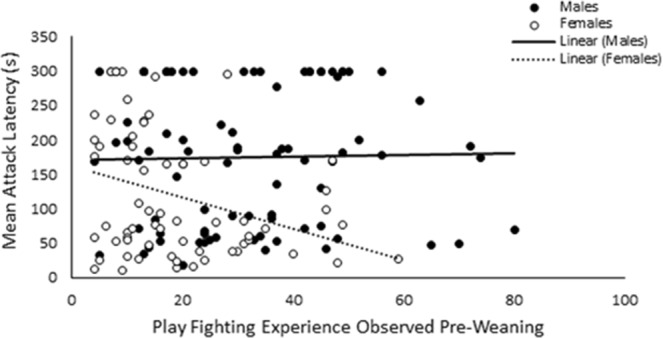
Correlations between mean attack latency and the total number of successful play fights observed pre-weaning for both male and female individuals. The solid and dashed lines indicate the linear line of best fit for males and females respectively.

Socialised residents were found to have a significantly shorter attack latency than control residents (socialised: 187.60 ± 13.12 s, control: 127.74 ± 9.86 s; χ^2^_1_ = 11.68, *p* < 0.001), while females were also quicker to attack the intruder than males (males: 174.18 ± 10.78 s, females: 119.16 ± 11.66 s; χ^2^_1_ = 16.93, *p* < 0.001). Play fighting did not directly influence latency to attack (χ^2^_1_ = 1.205, *p* = 0.272), although despite a non-significant interaction effect between play and sex (χ^2^_1_ = 2.86, *p* = 0.091), females with higher levels of play fighting experience tended to attack the intruder more quickly (see Fig. 9). Further analysis revealed a significant negative correlation between play fighting experience and average attack latency in females (Spearman’s rank correlation; *r*_*s*_ = −0.31, *D.F* = 64, *p* = 0.013), while this trend was not observed in males (Spearman’s rank correlation; *r*_*s*_ = 0.05, *D.F* = 84, *p* = 0.670). Aggressiveness, as indicated by RI testing, has previously been demonstrated to remain consistent over time^57^ and during this experiment individual attack latency between testing days was found to be significantly correlated (Spearman’s rank correlation; *r*_*s*_ = 0.56, *D.F* = 147, *p* < 0.001). Attack latency was however found to be shorter in the second RI test compared to the first (χ^2^_1_ = 12.08, *p* < 0.001).

## Discussion

The current study hypothesised that males would perform more social play than females during the pre-weaning period, allowing them to develop the social skills required for later-life interactions as aggression between adult males is potentially costly. In line with this prediction, males were found to engage in more pre-weaning play fights than females, while also performing more additional piglet directed play, such as chasing and mounting. Females however were found to direct more attention towards the sow than males. Furthermore it was predicted that socialised individuals would receive more pre-weaning play fighting experience than their control counterparts. Contrary to this prediction, socialised and control individuals did not differ in the amount of social play fighting experienced, although socialised individuals directed approximately a third of their inter-piglet play towards non-littermates. Control individuals were also found to perform more sow directed play than those experiencing socialisation. Despite not differing in their play fighting experience, socialised individuals were quicker to attack an inferior intruder during resident intruder testing. Additionally, females were found to be more aggressive than males and showed a significant correlation between pre-weaning play fighting experience and post-weaning aggression.

These findings support the hypothesis that social play behaviour is sexually dimorphic in species were males and females differ in their later-life social environment^40,43,44^ (but see^46^). Previously, findings regarding the effect of sex on piglet play behaviour have been divided. In agreement with Dobao *et al*.^40^ and Brown *et al*.^56,62^ we observed that males performed higher amounts of social play (including play fighting) than females. Furthermore, given the confinement of the sow, sow directed play could be an indicator of object play, or even locomotor play, which would support the sex difference in early-life play observed by Rauw^55^ and D’Eath & Lawrence^13^. The division of general categories of play (i.e. social, locomotor, and object), into more discrete behavioural activities, (i.e. play fighting, nudging, sow climbing) therefore appears to be important for the detection of sex-dependant variation. For example, Newberry *et al*.^30^ reported no effect of sex on the frequency of play markers when observed together, although males where observed to perform shoving and mounting behaviours more frequently than females^62^.

Despite being exposed to a more complex social environment, socialised piglets did not differ from controls regarding the amount of play fighting or additional piglet directed play they engaged in. We confirmed that socialised piglets engaged in play with non-littermates, with a tendency for additional piglet directed play with non-littermates to increase over time, despite showing higher levels of play towards littermates overall. These interactions with non-littermates are likely to serve an important role in the development of later-life social skills. Control individuals were found to engage in more sow-related interactions than socialised individuals, which may be due to piglets needing to find an alternative outlet for social behaviour given the reduced number of play partners. Stolba^63^ observed that the proportion of time grower piglets devoted to social activities was greater in barren environments, suggesting that a lack of interesting substrate can lead to the redirection of interest towards other group members. Alternatively, socialised piglets may have redirected a proportion of sow directed play towards the non-related sow.

Play fighting behaviour, additional piglet directed play, and sow directed play were all found to be highly concordant across the pre-weaning observation period, regardless of treatment, demonstrating individual consistency in play behaviour over time. Consistency in individual play motivation is further supported by the findings of Brown *et al*.^56^, who reported a significant correlation between the amount of social play piglets performed pre- and post- weaning. It appears that piglets that initially perform high levels of social play continue to do so throughout their early life development. Additionally, no clear pattern in the amount of social play performed across the observation period was apparent, further suggesting that early-life social play is a consistent aspect of animal personality^61^.

As has previously been reported^24,57,60^, resident individuals from both treatments were quicker to attack the intruder on the second day of testing. This may due to improved familiarity with the situation or increased aggressive motivation due to winner effects resulting from the previous RI test^64^. Consistent with the findings of D’Eath^24^, socialised residents were found to show a significantly shorter attack latency than controls. Additionally, play fighting experience was found to negatively correlate with attack latency in female pigs, suggesting play fighting experience may be linked to an increase in aggressiveness. However, no such correlation was observed in male individuals.

These findings suggest that, in addition to the play fighting experience of females, a further aspect of the socialisation treatment may be responsible for improving the assessment of social situations in both sexes. For example, the presence of non-littermates increases the variety of potential play partners an individual has access to, which in turn leads to variation in the range of social situations an individual can experience. Play can quickly lead to serious aggression if rules are not followed, however rules during play tend to be made on a here-and-now basis and can vary depending on the age, sex, and relation of the participants^65,66^. As such, experiencing a wider-range of play partners may require individuals to evaluate, and adapt to, a greater number of unique social contexts.

Additionally,  pre-weaning socialisation provides an opportunity for individuals to interact with non-littermates for the first time in a socially forgiving environment. Alternatively, the increase in available space caused by the socialisation treatment may have improved the quality of social play performed, despite piglet density remaining unchanged. Either way, the decreased attack latency of socialised pigs may indicate an individual’s improved recognition and understanding of agonistic situations. Further investigations into the effect of early-life play experience on behaviour during later-life agonistic encounters is currently on-going by our research group.

D’Eath^24^ found that socialised pigs were quicker to begin fighting in a group-mixing scenario and subsequently concluded hierarchy formation more quickly than controls. Additionally, fight duration was significantly reduced in the socialised treatment, supporting subsequent findings that suggest pre-weaning socialisation leads to a reduction in aggression post-weaning^25–27^.

Contrary to several previous reports^13,57,60,67^, females were found to be significantly more aggressive than males. Similar to early-life social play experience, sex differences in aggression are likely to be the result of the sexually dimorphic social environments commonly observed in free-ranging adult pigs. As is often the case in polygynous ungulates, the dispersal of wild boar is typically male-biased, leaving social groups comprising of closely-related females^68^. While female boar are facultative cooperative breeders and benefit from the retention of female offspring, fluctuations in mother-daughter social relations can occur throughout the year^67^. For example, periods such as the final stage of pregnancy/early lactation, and immediately after weaning, often coincide with periods of social instability in ungulates^68^. In the case of wild boar, sows typically isolate themselves from other group members during this period in order to focus on feeding piglets and ensuring a strong familial bond^16^, before returning to their social group approximately 2 weeks after farrowing^28,29^. This suggests that the cost of aggression is low in females as dominance relationships are only weakly enforced and competitors are often closely related. As such, females may be inclined to take more risks than males when engaging others in agonistic encounters.

Adult males on the other hand only associate with such social groups when sows are in heat^42^, and typically avoid each other outside of the mating session^69^. After joining a social unit (often referred to as a sounder), the adult male will proceed to chase off any remaining males over a year old and fight with any rival pigs who may try to gain access to the females^42,69,70^. As such, dominant male boars are reported to participate in more mating than subordinate males^71–73^. In order to establish dominance, boars fight head on, pushing each other with great force and attempting to slash each other with their tusks^70,73,74^. This form of aggression can be highly costly to both individuals in terms of both injury and losing individuals in terms of reduced reproductive success. It may therefore be beneficial for males to delay the performance of damaging aggression in agonistic situations until they have made a thorough assessment of their opponent, regardless of previous experience.

## Conclusion

Consistent with the sexual dimorphism hypothesis, males and females were found to show clear differences in their early-life social play experience. Contrary to our predictions, early-life socialisation did not result in increased play fighting behaviour, however females were found to have a shorter attack latency than males suggesting a sex difference in costs and benefits of later-life aggression. Furthermore, female play experience was negatively correlated with attack latency. This suggests that while socialisation may improve an individual’s capacity to perform appropriate social behaviours within an agonistic setting, the means through which this is achieved is dependent upon the sex of the individual.

## Supplementary information

Supplementary material

## Data Availability

The datasets generated and analyzed during this current study are available from the corresponding author on reasonable request.
