# Supplementary material for: Socialisation and its effect on play behaviour and aggression in the domestic pig (Sus scrofa)
Source: Sci Rep. 2019 Mar 12;9:4180. doi: 10.1038/s41598-019-40980-1 (PMC6414639; doi:10.1038/s41598-019-40980-1)
Supplement: Supplementary file 1 — Supplementary material [file 41598_2019_40980_MOESM1_ESM.pdf]

Supplementary Material: Socialisation and its effect on play behaviour and aggression in the domestic pig (*Sus scrofa)*

Jennifer E. Weller, Irene Camerlink, Simon P. Turner, Marianne Farish, Gareth Arnott

Supplementary Table S1 – Ethogram used to identify social play behaviour during video play back. Observed play behaviours were broken down into three categories, focusing on play fighting behaviour, additional piglet directed behaviour, and sow directed behaviour.

Behaviour	Description	Reference
**Play Fighting**		
Play Fighting Invite	Initiator piglet performs rapid face-to-face pushing actions directed at a target piglet. Pushing is often repetitive and performed in an energetic manner. Does not include deliberate/accidental shoving when the target pig is inhibiting initiator locomotion. All pushing and shoving under the udder is ignored during suckling bouts.	1,2
Success	Target piglet responds to the initiator piglets 'invite' by pushing back and engaging in a play response. Play occurs as both individuals push towards one another, with occasional head knocking and biting attempts. (See Accept Play).	Defined for this study
Failure	Initiator is unsuccessful at eliciting a play response from the target individual. Target piglet either turns its head/body away from the initiator piglet, moves away without further reaction, or does not give any noticeable response to the initiator piglet's attempts to play. (See Reject Play).	Defined for this study
Accept Play Fighting	Target piglet responds to initiator piglets invite by pushing back against the head of the initiator. This results in both individuals pushing towards one another, with occasional head knocking and biting attempts. (See Success).	Defined for this study
Reject Play Fighting	Target piglet does not response to initiator piglet. Target piglet either turns its head/body away from the initiator piglet, moves away without further reaction, or does not give any noticeable response to the initiator piglet's attempts to play. (See Failure).	1,2
Third Party Interaction	A third piglet attempts to join a pre-occurring play fight by either pushing one or both of the currently participating piglets. This interaction either results in the play behaviour finishing, one piglet being displaced from the play fight, or the continuation of the fight with a third member.	Defined for this study
**Additional Piglet Directed Play**		
Nudge	The snout of the initiator is used to make forceful contact with another piglet's body. This does not include face-to-face contact (See Invite Play). Does not include pushing past other pigs restricting passage during locomotion or joining a resting pile of piglets. Additionally, includes climbing attempts made from the side or front of the target piglet.	1,2
Mount	Initiator piglet attempts to place both front feet on the back of the target piglet. The attempt must be made from behind and both of the initiators front feet must leave the ground.	3
Chase	Initiator piglet follows the locomotory movement and direction of a target piglet intensely. I.e. if the target piglet runs and turns right, so does the initiator piglet.	1
**Sow Directed Play**		
Naso-Naso Contact	Initiator piglet touches its snout to the snout/face of its own sow. This can be invoked either by the initiator piglet or by the sow.	4
Climbing	Initiator piglet uses its front feet to elevate itself onto the body of its own sow. At least two feet must be off the ground and in contact with the sow. Activity under the udder during suckling is ignored (i.e. climbing to reach a preferred teat etc.).	1

1. Martin, J. E., Ison, S. H. & Baxter, E. M. The influence of neonatal environment on piglet play behaviour and post-weaning social and cognitive development. *Appl. Anim. Behav. Sci.* **163**, 69-79 (2015).

2. Brown, S. M., Klaffenböck, M., Nevison, I. M. & Lawrence, A. B. Evidence for litter differences in play behaviour in pre-weaned pigs. *Appl. Anim. Behav. Sci.* **172**, 17-25 (2015).

3. Bolhuis, J. E., Schouten, W. G., Schrama, J. W. & Wiegant, V. M. Behavioural development of pigs with different coping characteristics in barren and substrate-enriched housing conditions. *Appl. Anim. Behav. Sci.* **93**, 213-228 (2005).

4. Blackshaw, J., Swain, A., Blackshaw, A., Thomas, F. & Gillies, K. The development of playful behaviour in piglets from birth to weaning in three farrowing environments. *Appl. Anim. Behav. Sci.* **55**, 37-49 (1997).
